# Uterus-preserving management of a high-flow uterine artery pseudoaneurysm with arteriovenous fistula following cesarean section: a case report

**DOI:** 10.1016/j.crwh.2026.e00821

**Published:** 2026-05-22

**Authors:** Benedict Krischer, Ruben Lopez Benitez, Ivo Fähnle, Stephanie Verta, Markus Hodel

**Affiliations:** a Lucerne Cantonal Hospital, Department of Gynecology & Obstetrics, Kantonsspital 37, 6004 Lucerne, Switzerland; b Lucerne Cantonal Hospital, Department of Interventional Radiology, Kantonsspital 37, 6004 Lucerne, Switzerland; c Lucerne Cantonal Hospital Sursee branch, Department of Gynecology & Obstetrics, Spitalstrasse 16A, 6210 Sursee, Switzerland

**Keywords:** Cesarean section, Secondary postpartum hemorrhage, Uterine artery pseudoaneurysm, Arteriovenous fistula, Doppler ultrasound, Embolization

## Abstract

Postpartum uterine vascular lesions are a rare but important cause of secondary postpartum hemorrhage. This report describes the case of a 29-year-old woman presenting five weeks after repeat cesarean section with recurrent vaginal bleeding. Transvaginal color Doppler ultrasound demonstrated a 2.7 cm anechoic lesion adjacent to the cesarean scar with marked hypervascularization and a high-flow pattern (peak systolic velocity up to 310 cm/s, low resistance indices) with pulsatile venous outflow, suggestive of a combined uterine artery pseudoaneurysm and arteriovenous fistula. Computed tomography CT angiography confirmed a pseudoaneurysm of the uterine artery with early venous drainage via the right uterine vein.

Selective embolization of the right uterine artery resulted in a substantial but incomplete reduction in flow. Follow-up Doppler ultrasound demonstrated persistent perfusion, and repeat embolization was required to treat residual feeders from both uterine and ovarian circulation. Serial Doppler assessment showed progressive reduction in flow velocities and lesion size, with complete sonographic resolution after four months. One year later, the patient had another successful pregnancy.

This case illustrates that high-flow uterine vascular lesions following cesarean section may involve complex collateral supply and require repeat embolization. Transvaginal color Doppler ultrasound is central not only for initial diagnosis but also for monitoring treatment response. Persistent flow after initial embolization does not necessarily indicate treatment failure but may reflect collateral reperfusion amenable to targeted re-intervention. Uterus-preserving management can result in complete resolution and allow subsequent pregnancy.

## Highlights

• Uterine vascular lesions can show high-flow, low-resistance Doppler patterns.
• A pseudoaneurysm may coexist with AV fistula and show a typical high-flow pattern.
• CT angiography shows venous drainage and collateral supply.
• A successful pregnancy can be achieved even after repeat embolization.

## 1 Introduction

Rates of cesarean sections (CS) differ markedly between countries, but can be as high as 56% (in Brazil). The European average is around 20–27% [1]. With increasing CS rates, awareness of rare but potentially life-threatening postoperative complications is essential. Uterine artery pseudoaneurysm (UAP) is an uncommon but potentially dangerous cause of secondary (i.e. delayed) postpartum hemorrhage (PPH) [2]. It typically develops secondary to vessel injury, for example at the site of hysterotomy. Pseudoaneurysms are lesions caused by disruption of the vessel wall with persistent communication to the parent vessel, lacking the original three-layer artery wall [3]. Due to turbulent flow, they can expand. A rupture can lead to catastrophic hemorrhage.

CS is one of the most frequently reported antecedent procedures for UAP. In a review by Isono et al., CS accounted for 47.4% of reported cases, followed by dilation and curettage (D&C) and myomectomies [4]. In a large single-center case series, Baba et al. described an incidence of 0,3% of deliveries [5].

Color Doppler ultrasound is the first-line diagnostic modality, while computed tomography (CT) and angiography can support the diagnosis and guide management [4]. Treatment options depend on clinical presentation and consideration of future fertility [3], [6]. Most patients are treated by transcatheter arterial embolization. Takahashi et al. argue that asymptomatic cases with small UAP – in their case series <15 mm – might be managed expectantly [6]. In emergency cases with hemodynamic instability, hysterectomy is the last resort.

UAP should be distinguished from iatrogenic arteriovenous fistulas (AVF), i.e. a direct arterio-venous connection secondary to a vessel injury during D&C or CS [7], [8]. The distinction, however, can clinically be challenging as the two entities have a common etiology and similar presentation on Doppler ultrasound, and can even coexist as part of a spectrum of postoperative lesions [9], [10], [11], [12], [13]. Nevertheless, it is important to distinguish between UAP and AVF: while both can lead to hemorrhage and the armamentarium of management is the same, the underlying pathophysiology is different and therefore the distinction may influence course and prognosis.

The following case describes a post-cesarean high-flow uterine vascular lesion adjacent to the cesarean scar, combining a pseudoaneurysm with an arteriovenous fistula. Serial Doppler follow-up documented persistent flow after initial embolization and progressive hemodynamic normalization after repeat embolization. The differentiation of entities will be discussed as well as the choice of management in case of coexistence.

## 2 Case Presentation

A 29-year-old woman (G4P1) underwent repeat cesarean section at 39 + 0 weeks of gestation. She had a history of a previous cesarean section. The repeat cesarean section was uncomplicated except for an estimated blood loss of approximately 1000 mL.

**Fig. 1:**
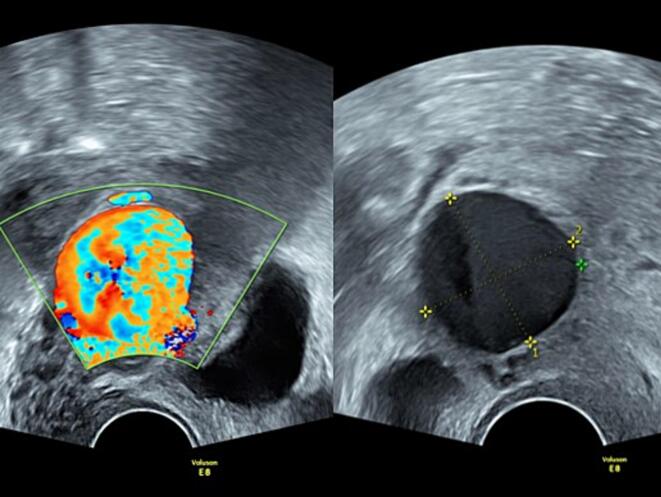
Initial presentation 5 weeks post-partum. Left: strong aliasing on Doppler in a round structure. Right: 27x27x30mm diameter round hypoechoic structure on B-mode.

Five weeks postpartum, she presented to the emergency department with vaginal bleeding, having soaked three maternity pads within a short period of time. She was hemodynamically stable and her hemoglobin level was 116 g/L. Transvaginal ultrasound revealed an anechoic round lesion measuring approximately 2.7 × 2.7 cm adjacent to the right cervical region at the level of the cesarean scar (Fig. 1).

**Fig. 2:**
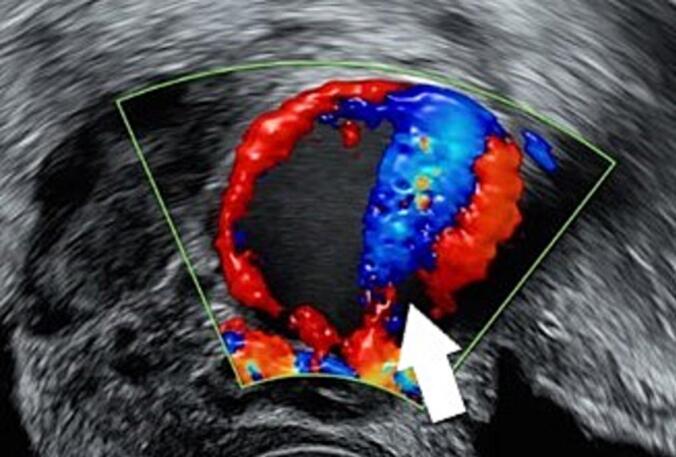
Arterial jet (arrow) entering UAP, blood flow reflecting from the opposite wall end draining close to the entry point.

At follow-up two days later, the patient reported recurrent episodes of increased vaginal bleeding “with blood stains on the floor”. Color Doppler imaging demonstrated a high-velocity jet entering the lesion from the right uterine artery (Fig. 2). The intralesional flow then coursed caudally along the wall, with venous outflow most consistent with drainage into the right uterine vein. The Doppler assessment demonstrated marked high-flow hemodynamics, with peak systolic velocity up to 310 cm/s, end-diastolic velocity 204 cm/s, resistive index (RI) and pulsatility index (PI) of 0.34. CT angiography confirmed an AVF of the uterine vessels adjacent to the cesarean scar, associated with a pseudoaneurysm measuring approximately 30 × 27 × 27 mm and early venous drainage through the right uterine vein and internal iliac venous system.

**Fig. 3:**
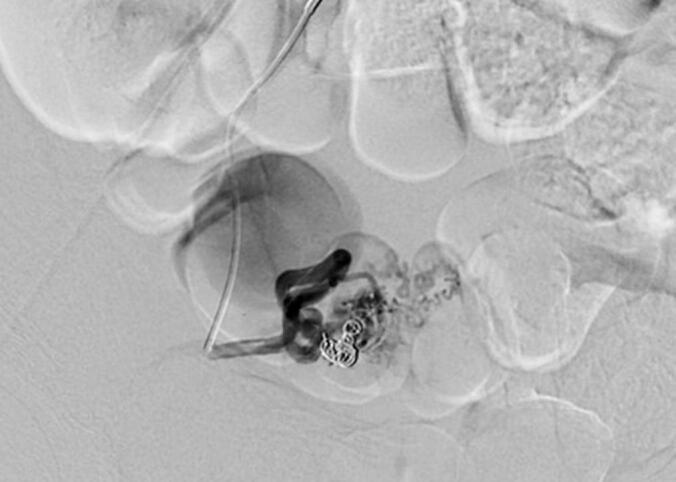
Image from first embolization: coils being placed in the feeding vessel.

Because of the lesion size, slow persistent bleeding, high-flow pattern and the patient's wish for fertility preservation, interventional treatment was pursued. One day later, selective embolization of the pseudoaneurysm of the right uterine artery was performed using 0,018″ microcoils (IDC-coils, Boston Scientific, USA) and cyanoacrylate/lipiodol (Glubran, Italy; Guerbet, France), resulting in a major but incomplete reduction in flow (Fig. 3).

Follow-up ultrasound another three days later showed persistent perfusion with peak systolic velocity of 154 cm/s and end-diastolic velocity of 110 cm/s, with a residual lesion size of 17 × 16 mm. A second embolization was therefore undertaken. Angiography demonstrated residual perfusion from small branches of the right uterine artery and additional collateral supply from the right ovarian circulation. These feeders were embolized using again 0,018″ microcoils and cyanoacrylate/lipiodol, until significant flow reduction of the UAP was achieved.

Subsequent inpatient ultrasound follow-up showed ongoing but progressively reduced perfusion. On discharge three days later, the lesion measured 17 × 13 × 17 mm and showed marginal thrombosis, with peak systolic velocity 141 cm/s and end-diastolic velocity 94 cm/s.

On outpatient follow-up three weeks after the second intervention, the lesion diameter had decreased to 12.5 mm, with further hemodynamic improvement: peak systolic velocity 74 cm/s, end-diastolic velocity 41 cm/s, resistive index 0.45 and an estimated pulsatility index of ∼0.63.

The patient remained clinically stable and required no surgical intervention. Serial outpatient ultrasound follow-up demonstrated continued regression. At follow-up about 4 months after the second intervention the lesion was no longer detectable sonographically. A subsequent pregnancy in the following year resulted in preterm repeat cesarean section at 35 + 6 weeks of gestation, with an uneventful perioperative and postpartum course and no evidence of recurrence.

## 3 Discussion

This case illustrates a rare but clinically important cause of secondary postpartum hemorrhage after cesarean section: a high-flow uterine vascular lesion adjacent to the cesarean scar combining a pseudoaneurysm with an arteriovenous fistula. Both pseudoaneurysms and AVF share a common etiology: uterine trauma from cesarean section, curettage, myomectomy, or other surgical interventions [4], [14]. This shared pathophysiology explains why they can occur simultaneously.

UAP presents with a round hypoechogenic structure. Doppler findings include turbulent flow with aliasing and a “to-and-fro” waveform at the neck of the lesion [11]. In contrast, the presence of venous outflow with pulsatility and markedly reduced downstream resistance supports arteriovenous communication [15]. In the present case, the combination of a large lesion at the hysterotomy site, extremely high flow velocities and a venous drainage on Doppler and CT strongly suggested a pseudoaneurysm with associated AVF.

While pseudoaneurysms carry a risk of rupture [4], the need for repeat embolization appears to be more closely related to high-flow shunting and collateral supply than to the distinction between AVF and UAP itself [7].

An aliasing on color flow Doppler can be seen in other entities as well, such as enhanced myometrial vascularity (EMV) or arteriovenous malformations (AVM), but hypo−/anechoic lesion in B-mode and a high PSV hint at UAP. Table 1 gives an overview of the presentation of similar lesions. Importantly, the terms AVM and AVF are frequently conflated in the gynecologic literature, creating overlaps in literature review.

**Table 1:** Overview of differential diagnosis of uterine vascular lesions.

Feature	AVM – Arteriovenous malformation	AVF - Arteriovenous fistula	EMV - Enhanced myometrial vascularity	UAP - Uterine artery pseudoaneurysm
Etiology	Congenital	Iatrogenic	Post-pregnancy, associated with RPOC	Iatrogenic
Underlying patho-physiology	Abnormal embryologic development of primitive vessels, with arteriovenous connections bypassing capillary bed. Involves multiple pelvic vessels. No antecedent trauma/ pregnancy	Direct fistulous communication between artery and vein after vascular injury. Common collaterals.	Potentially part of normal uterine involution. Hypervascularity of placental bed	Arterial wall defect with extraluminal blood cavity communicating with artery
Typical clinical presentation	Menorrhagia or asymptomatic incidental finding on ultrasound.	Intermittent or heavy vaginal bleeding after uterine instrumentation	Post-pregnancy bleeding or incidental finding; often after miscarriage, abortion, or RPOC	Delayed/sondary postpartum or post-procedural hemorrhage; often sudden and potentially massive bleeding
Ultrasound presentation	Intense vascular tangle with tortuous vessels; high-velocity (PSV >96 cm/s), low-resistance flow (RI 0.25–0.55); mosaic pattern with turbulent flow; spectral broadening; venous flow shows arterial pattern	Similar to AVM	Tortuous dilated myometrial vessels, may be diffuse; PSV ≥20 cm/*sec*; low-resistance pattern; less intense than true AVM; may have associated RPOC	Round/ovoid anechoic structure; “yin–yang” color pattern, to-and-fro waveform at the neck; high peak velocity in feeding vessel
Course & management	Does not regress spontaneously. Might be difficult to treat due to multi-vessel involvement. Often requires repeat interventions Stable: expectant or medical (e.g. hormonal therapy, TXA); symptomatic/high-flow: embolization; rarely surgery	Occasionally resolve spontaneously. Expectant management in selected cases, if stable; otherwise embolization or surgical.	Usually expectant management; resolution accelerated after RPOC expulsion	First-line: embolization; small cases may resolve spontaneously; surgery if unstable or embolization fails

The majority of cases of secondary postpartum hemorrhage after cesarean section are related to infection [2], [16]. In cases of severe or recurrent bleeding, Doppler ultrasound may be valuable to identify other causes such as uterine vascular lesions.

Color Doppler ultrasound was central in this case not only for diagnosis but also for longitudinal monitoring. The lesion initially demonstrated extremely high peak systolic and diastolic velocities with very low resistance. After the first embolization, persistent flow remained, reflecting incomplete occlusion and collateral reperfusion. This is plausible in view of the complex pelvic arterial network and is supported by prior reports describing redistribution or collateral supply requiring repeat or bilateral embolization. The second embolization confirmed residual feeders from both uterine and ovarian circulation and resulted in definitive obliteration.

Management of postpartum uterine vascular lesions must be individualized. In hemodynamically stable patients, conservative management has been reported for selected uterine AVM/ AVF or some pseudoaneurysms. Timmermann et al. propose that patients with AVM/AVF with a PSV <40 cm/s could be managed expectantly while a PSV >80 cm/s indicates more dangerous lesions and warrants intervention [15].

In the review by Isono et al., transcatheter arterial embolization was used in the overwhelming majority of reported UAP cases and is widely regarded as the uterus-preserving treatment of choice. Cooper et al., Wu and others describe cases with UAP which required bilateral embolization [3], [5], [17]. Some cases show similarities to the present case, with rebleeding due to feeding from collateral arteries and higher success rates after bilateral embolization. This raises the question of whether direct bilateral embolization might be more advantageous, as fertility preservation after bilateral embolization seems favorable.

Complete embolization of an aneurysmatic lesion is preferable over an incomplete embolization with significant aneurysmatic blood flow reduction.

However, if a complete aneurysmatic embolization is technically not possible, in most cases, reduced blood flow velocity inside the aneurysm after embolization may result in an aneurysm thrombosis after several days or weeks. Reduced blood flow, new blood flow turbulences and the natural thrombotic mechanisms facilitate the thrombotic process [18].

As long as the bleeding is controlled, expectant management seems reasonable for selected cases of uterine vascular lesions. Due to the rupture risk, UAP is generally considered less stable and often warrants prompt treatment. As shown by the present case, persistent or recurrent flow after embolization does not necessarily indicate failure but may reflect collateral reperfusion requiring targeted re-intervention. In serial Doppler measurements, progressive reduction in PSV and increase in RI paralleled clinical and morphological resolution.

The later successful pregnancy and repeat cesarean delivery without recurrence are reassuring and consistent with prior reports that uterus-preserving management can allow future reproduction.

## 4 Conclusion

Post-cesarean uterine vascular lesions should be considered in women presenting with secondary postpartum hemorrhage and no signs of infection. A combination of transvaginal color Doppler ultrasound and CT angiography can accurately characterize lesion morphology and flow pattern. Depending on initial presentation, size and characterization of the lesion, further management is planned. Doppler ultrasound is valuable in guiding the need for re-intervention and post-intervention surveillance. Fertility can be preserved even after repeat embolization.

## Contributors

Benedict Krischer contributed to patient care, conception of the case report, drafting the manuscript, undertaking the literature review and revising the article critically for important intellectual content.

Ruben Lopez Benitez contributed to patient care and revising the article critically for important intellectual content.

Ivo Fähnle contributed to patient care and revising the article critically for important intellectual content.

Stephanie Verta contributed to patient care and revising the article critically for important intellectual content.

Markus Hodel contributed to patient care and revising the article critically for important intellectual content.

All authors approved the final submitted manuscript.

## Patient consent

Written informed consent was obtained from the patient for publication of this case report and accompanying images.

## Provenance and peer review

This article was not commissioned and was peer reviewed.

## Funding

No funding from an external source supported the publication of this case report.

## Declaration of competing interest

The authors declare that they have no competing interest regarding the publication of this case report.
